# Shedding light on iron nutrition: exploring intersections of transcription factor cascades in light and iron deficiency signaling

**DOI:** 10.1093/jxb/erae324

**Published:** 2024-08-08

**Authors:** Ksenia Trofimov, Samriti Mankotia, Mary Ngigi, Dibin Baby, Santosh B Satbhai, Petra Bauer

**Affiliations:** Institute of Botany, Heinrich-Heine-University, D-40225 Düsseldorf, Germany; Department of Biological Sciences, Indian Institute of Science Education and Research (IISER), Mohali, SAS Nagar, Punjab 140406, India; Institute of Botany, Heinrich-Heine-University, D-40225 Düsseldorf, Germany; Cluster of Excellence on Plant Science (CEPLAS), Heinrich-Heine-University, D-40225 Düsseldorf, Germany; Institute of Botany, Heinrich-Heine-University, D-40225 Düsseldorf, Germany; Department of Biological Sciences, Indian Institute of Science Education and Research (IISER), Mohali, SAS Nagar, Punjab 140406, India; Institute of Botany, Heinrich-Heine-University, D-40225 Düsseldorf, Germany; Cluster of Excellence on Plant Science (CEPLAS), Heinrich-Heine-University, D-40225 Düsseldorf, Germany; National Institute of Science Education and Research, India

**Keywords:** bHLH, biomolecular condensate, blue light, BTS, circadian clock, FIT, HY5, iron, long-distance signaling, SR45

## Abstract

In the dynamic environment of plants, the interplay between light-dependent growth and iron nutrition is a recurring challenge. Plants respond to low iron levels by adjusting growth and physiology through enhanced iron acquisition from the rhizosphere and internal iron pool reallocation. Iron deficiency response assays and gene co-expression networks aid in documenting physiological reactions and unraveling gene-regulatory cascades, offering insight into the interplay between hormonal and external signaling pathways. However, research directly exploring the significance of light in iron nutrition remains limited. This review provides an overview on iron deficiency regulation and its cross-connection with distinct light signals, focusing on transcription factor cascades and long-distance signaling. The circadian clock and retrograde signaling influence iron uptake and allocation. The light-activated shoot-to-root mobile transcription factor ELONGATED HYPOCOTYL5 (HY5) affects iron homeostasis responses in roots. Blue light triggers the formation of biomolecular condensates containing iron deficiency-induced protein complexes. The potential of exploiting the connection between light and iron signaling remains underutilized. With climate change and soil alkalinity on the rise, there is a need to develop crops with improved nutrient use efficiency and modified light dependencies. More research is needed to understand and leverage the interplay between light signaling and iron nutrition.

## Teaser

Light and iron are both essential for plant functioning. Here, we review recent discoveries explaining the intricate interconnections involving regulatory components and systemic signals of iron and light signaling pathways.

## Introduction

Iron is among the most abundant elements in the Earth’s crust, where it is mostly present in the form of oxidized ferric iron in non-soluble iron oxide and oxyhydroxide compounds of mineral rocks, hence representing large stores of non-bioavailable iron. The levels of atmospheric oxygen substantially increased due to the emergence of oxygen-producing photosynthetic organisms >2 billion years ago. This so-called Great Oxidation Event had profound effects on the Earth’s environment and the evolution of life. It significantly impacted the bioavailability of iron, and the resulting race for iron has become a strong selection pressure for the new evolving life forms (Wade *et al.*, 2021). For terrestrial plants, iron bioavailability is heavily influenced by soil pH and moisture levels, which are in turn dictated by climatic conditions. The transition between dry and wet climates with mostly either alkaline or acidic soils occurs abruptly, leading to corresponding shifts between zones with bioavailable and non-bioavailable iron (Slessarev *et al.*, 2016). Global warming and anthropogenic activities may contribute to an alkalinization of soils, even though this phenomenon seems far more complex, involving additional factors such as radiation and soil depth (Sun *et al.*, 2023). Clearly, land plants live in an ever-increasing harsh environment with regard to mineral availability. The increase of alkaline soil and drought will also change vegetation and expose plants more frequently to direct sunlight. Hence, the strategies that plants use to deal with critically low amounts of iron in the rhizosphere, while also reacting to light, will be even more important with climate change.

Light is pivotal for plants as it facilitates biochemical redox and electron transfer reactions. Iron cofactor-containing enzymes and proteins (e.g. those utilizing heme–iron, iron–sulfur clusters, or free iron) play roles in nutrient assimilation (e.g. for reduction of carbon, nitrogen, and sulfur), and occur in many biochemical pathways in plants, particularly in mitochondria and chloroplasts. Photosynthesis is among the most noteworthy biochemical reactions that depends on iron and light. The importance of iron in light-controlled nutrient assimilation is explained by its high reactivity as a redox reagent to shuffle between ferric Fe^3+^ and ferrous Fe^2+^ states. One potential drawback for plant health is that ferrous iron is a strong Fenton reagent. In an oxygenated environment, Fenton reagents catalyze the reduction of oxygen species. As a result, various types of reactive oxygen species (ROS) can be generated in the presence of ferrous iron, including hydroxyl radicals, that are the most potent ROS (Le *et al.*, 2019). Oxidative stress also occurs in chloroplasts under high light or when the substrates for electron transfer are overloaded, as it is frequently the consequence of abiotic stresses linked with water deprivation (Choudhury *et al.*, 2017; Kanwar *et al.*, 2021). Hence, it is important for plants to adjust iron acquisition and homeostasis with light during plant growth and upon environmental stresses.

Plants have distinct iron and light signaling pathways, whereby recent studies uncover intricate connections between them. Yet limited research directly explores the significance of light in iron acquisition, which underscores the necessity for further investigation to develop sustainable, nutrient-efficient crops by focusing in particular on understanding and utilizing the interplay between light signaling and iron nutrition. Here, we summarize recent evidence indicating the multiple interconnections between light and iron signaling.

## Iron acquisition and allocation under iron deficiency

**Fig. 1.**
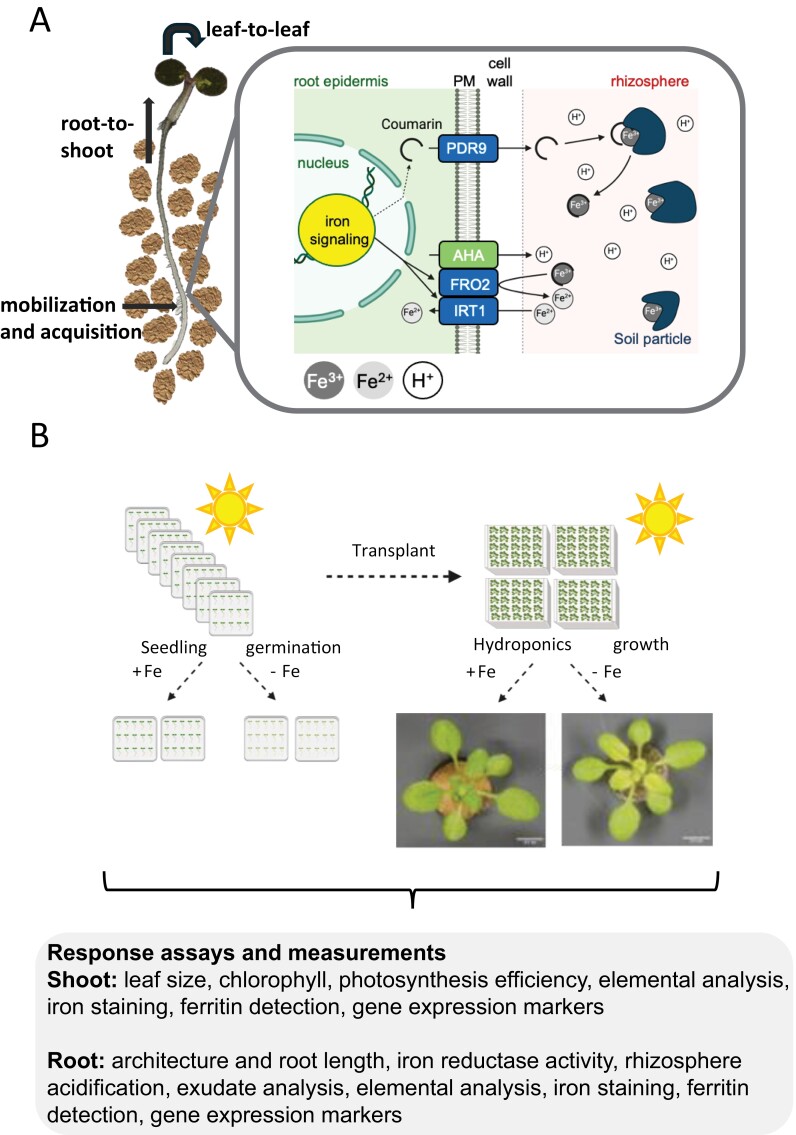
Overview of iron acquisition and allocation and iron deficiency response assays (example of *Arabidopsis thaliana*). (A) Iron is mobilized in the rhizosphere, acquired by roots, and transported from root to shoot. *A. thaliana* is a Strategy I plant, that secretes coumarins, acidifies the rhizosphere, reduces ferric iron (Fe^3+^), and takes up ferrous iron (Fe^2+^). PM, plasma membrane; FRO2, FERRIC REDUCTASE OXIDASE2; IRT1, IRON-REGULATED TRANSPORTER1. (B) Iron deficiency responses are studied by growing plants on agar plates or in hydroponic medium and exposing plants to iron-sufficient and -deficient conditions. Various responses can be qualitatively and quantitatively analyzed in shoots and roots. The figure has been created with Biorender.com. Iron-deficient and -sufficient plant growth phenotypes are shown for hydroponic growth.

Light can affect multiple aspects of iron homeostasis. To investigate light and iron signaling interconnections, it is important to understand the key processes of iron uptake and distribution in plants. Plants that experience iron deficiency can mobilize iron in the rhizosphere and increase their capacity to take up iron efficiently, and/or they can re-allocate iron within the plant (typical iron deficiency responses of seedlings are summarized in Fig. 1A).

Plants acquire iron primarily in the root differentiation zone above the root tip. They can mobilize iron in various ways in the rhizosphere depending on genetic and environmental factors. In the past, plants have been divided into two major groups. One group has been defined by the capacity to acidify the soil, and reduce and acquire ferrous iron (Strategy I, non-graminaceous plants, represented in Fig. 1A). The other group has been characterized based on secretion and uptake of phytosiderophores, which are chelators that form a complex with ferric iron (Strategy II, grasses) (Römheld and Marschner, 1986; Brumbarova *et al.*, 2015; Connorton *et al.*, 2017). Meanwhile, the general importance of coumarins and flavins has been clearly stated. These compounds are secreted under iron deficiency to enhance iron mobilization and reduction depending on ecological constraints of the habitat, such as pH, and on the species (Rodríguez-Celma *et al.*, 2011; Clemens and Weber, 2016; Lefèvre *et al.*, 2018; Li *et al.*, 2023). It has emerged that coumarins play a role in plant–microbe communication as microbes and endophytes play important roles in helping plants mobilize iron through microbial siderophores and plant growth-promoting molecules (Stassen *et al.*, 2021). Iron acquisition is also modulated by changing root cell barriers along the root (Zandi *et al.*, 2023; Grünhofer *et al.*, 2024) and altering root cell differentiation and root architecture (López-Bucio *et al.*, 2003).

In parallel to acquiring new iron, plants liberate iron from internal stores of roots and shoots. Iron is bound to cell wall material, such as pectin, and modifying pectin structure allows iron to accumulate (Zhu *et al.*, 2024). Iron is stored inside cell vacuoles, a process affected by altered vacuolar iron transport (Bastow *et al.*, 2018; Ram *et al.*, 2021). Iron stored in ferritin complexes, that are macromolecular assemblies for iron storage, or iron present in the electron transport chains in plastids and mitochondria can be released through autophagy, a form of targeted degradation of compartments under nutrient deficiency (Wan and Ling, 2022). Iron is also detected in the nucleolus (Roschzttardtz *et al.*, 2011). Plants control the amount of iron that is allocated to different plant parts. Iron is transported upwards in the inner stele of the root via xylem, dependent on transpiration, and from source to sink in the phloem. These types of allocation require different soluble iron-binding compounds and chelators (Schuler *et al.*, 2012; Kovács *et al.*, 2016).

Plant responses can be recorded by performing iron deficiency response assays which can provide evidence of the degree of iron deficiency and the responsiveness of plants to mobilize iron (Fig. 1B). Such assays have been informative to interpret phenotypes of mutants harboring defects in the acquisition and distribution of iron (Fig. 1B).

Overall, plants exhibit a multifaceted array of reactions in response to iron deficiency, aimed at fine-tuning their growth and physiology. These iron-dependent responses can be meticulously documented, allowing for thorough exploration of their interplay with other abiotic factors, such as variations in light quality.

## Iron deficiency response signaling

**Fig. 2.**
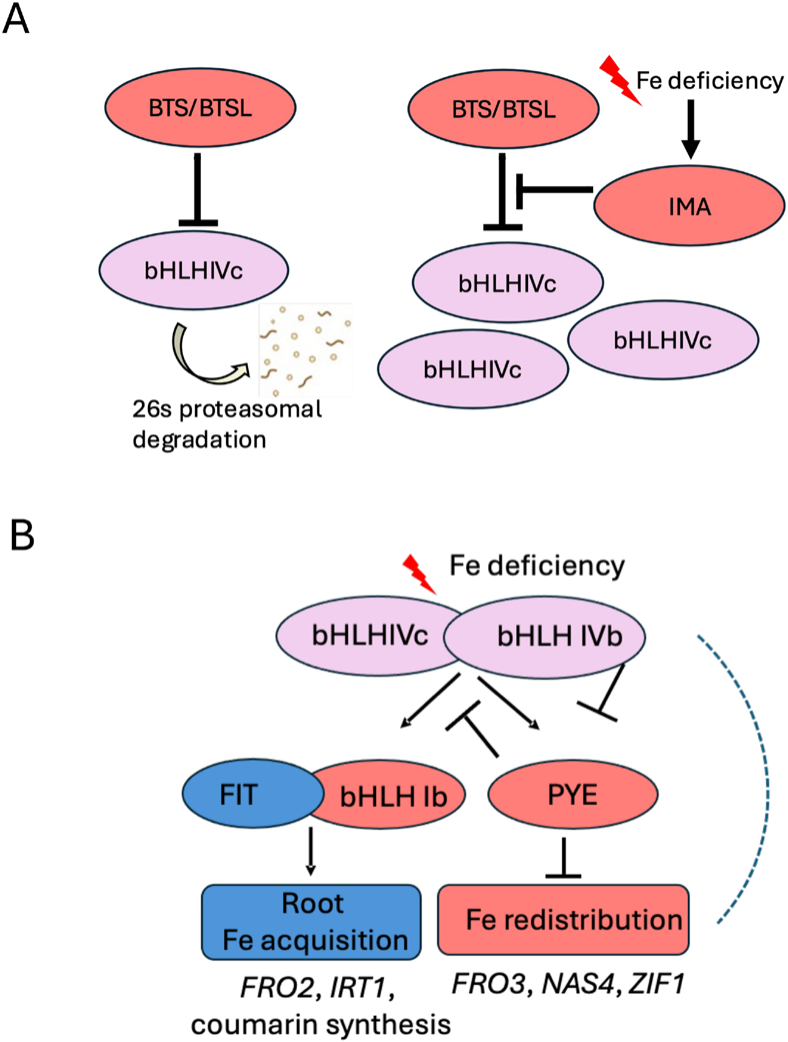
bHLH transcription factor cascade regulating plant iron homeostasis. The iron homeostasis pathway in plants is tightly regulated by bHLH transcription factors, E3 ligases, and other proteins. (A) E3 ligases, such as BTS (BRUTUS) and BTSL (BTS-like) proteins, can control the protein levels of bHLH IVc transcription factors by targeting them for proteasomal degradation. During iron deficiency, IMA (IRON MAN) proteins may repress BTS/L function by direct binding and serving as an alternative target for degradation, thereby increasing the abundance of bHLH IVc proteins. (B) Transcriptional cascade leading to iron deficiency responses. Interaction of bHLH IVc and IVb transcription factors leads to downstream targeting of another bHLH subgroup IVb member PYE (POPEYE) and the bHLH subgroup Ib (bHLH38, bHLH39, bHLH100, and bHLH101) for transcriptional regulation; for example, bHLH IVc plus URI (UPSTREAM REGULATOR OF IRT1)/bHLH121 leads to activation, while bHLH IVc plus bHLH11 leads to de-activation. Active PYE protein represses the expression of certain target genes, *FRO3* (*FERRIC REDUCTASE OXIDASE3*), *NAS4* (*NICOTIANAMINE SYNTHASE4*), *ZIF1* (*ZINC FACILITATOR1*), and *BHLH IB*, which causes iron redistribution. On the other hand, bHLH Ib transcription factors activate root iron acquisition by heterodimerization with a central regulator, FIT (FER-LIKE IRON DEFICIENCY-INDUCED TRANSCRIPTION FACTOR), leading to the activation of root-induced genes *FRO2* (*FERRIC REDUCTASE OXIDASE2*) and *IRT1* (*IRON-REGULATED TRANSPORTER1*). The dashed line indicates that regulation of target genes by bHLH IVc and IVb is also possible. The color code indicates distinct patterns of gene regulation: ‘blue’ color, iron deficiency-induced and FIT-dependent, acting primarily in seedling roots; ‘red’ color, iron deficiency-induced, but not dependent on FIT, acting in seedling roots and shoots; ‘rose’ color, not induced by iron deficiency, not dependent on FIT, acting in seedling roots and shoots. References are mentioned in the text. The figure has been created with Biorender.com.

As in response to light, plant responses to iron supply are triggered by signaling systems involving E3 ligases and basic helix–loop–helix (bHLH) transcription factors. Thus, numerous crossing links may exist due to genetic and biochemical interactions. Therefore, it is important to understand the players that regulate iron deficiency response signaling. When iron deficiency is sensed by plants, thousands of genes become differently regulated during the following 3 days. A small subset of iron deficiency-induced genes is directly involved in the acquisition and allocation of iron upon iron deficiency stress (Schwarz and Bauer, 2020; Schwarz *et al.*, 2020). These genes encode proteins for iron mobilization, iron transport, and iron chelation or storage, and they can be integrated in regulatory iron-responsive networks (Fig. 2). The remaining differentially expressed genes reflect the broad metabolic, hormonal, and physiological spectrum of plant responses to iron deficiency (Brady *et al.*, 2007).

Iron availability is sensed by plants; however, concrete iron sensor proteins still need to be identified and confirmed for plants. Among the candidates for sensing iron are the *Arabidopsis thaliana* proteins BRUTUS (BTS) and BTS-LIKE proteins BTSL1 and BTSL2, which are E3 ligases (Rodríguez-Celma *et al.*, 2019). Based on mutant studies, BTS/BTSLs can be classified as negative regulators of iron acquisition and homeostasis (Long *et al.*, 2010; Hindt *et al.*, 2017). They share some similarity in structure with the human iron-sensing F-box and leucine-rich repeat protein 5 (FBXL5), which is a substrate adaptor of an E3 ubiquitin ligase complex. FBXL5 stability and target protein interaction are controlled by oxygen and iron (Mayank *et al.*, 2019; Wang *et al.*, 2020). BTS/BTSL proteins possess hemerythrin domains for iron–oxygen binding, and their protein stability characteristics are linked with the presence of iron binding to this site (Selote *et al.*, 2015). A current model of the mode of action is that BTS/L proteins target the top layer of regulation (Li *et al.*, 2021; Lichtblau *et al.*, 2022) (Fig. 2A). This layer is represented by the bHLH transcription factors that activate the iron deficiency response, among them in particular the bHLH subgroup IVb and IVc transcription factors. For example, bHLH IVc transcription factors, such as bHLH034, bHLH104, bHLH105/ILR3, and bHLH115, promote iron deficiency responses (Zhang *et al.*, 2015; Li *et al.*, 2016; Liang *et al.*, 2017; Samira *et al.*, 2018; Tissot *et al.*, 2019). bHLH IVb factors, such as bHLH121/URI (Gao *et al.*, 2019; Kim *et al.*, 2019), can activate iron deficiency responses, or others such as bHLH11 (Liang *et al.*, 2017; Tanabe *et al.*, 2019; Li *et al.*, 2022) can deactivate these responses. In the absence of the promoting transcription factors, plants have reduced capabilities for iron uptake, while iron accumulates when they are overactivated (Liang, 2022). The interactions with BTS/L proteins might lead to ubiquitination and destabilization of the bHLH transcription factors. BTS/L proteins may be involved in iron signaling considering that their stability depends on the presence of iron cofactors. Clearly, more research is needed to confirm in plants whether and how BTS/L protein stability is iron dependent. The BTS/L–bHLH protein interaction can be counteracted by small proteins (~5 kDa) of the IRON MAN (IMA) family that are positive regulators of iron uptake (Li *et al.*, 2021; Lichtblau *et al.*, 2022; Peng *et al.*, 2022). A possible scenario is that IMA protein effectors can block the degradation of bHLH transcription factors by themselves becoming targets of ubiquitination via BTS/Ls (Li *et al.*, 2021) (Fig. 2A).

bHLH IVb and IVc transcription factors control a second level of bHLH proteins (Fig. 2B). They comprise two types, in *A. thaliana*, the subgroup IVb transcription factor POPEYE (PYE) (Long *et al.*, 2010) and the subgroup Ib transcription factors bHLH38, bHLH39, bHLH100, and bHLH101 (Wang *et al.*, 2007, 2013; Naranjo Arcos *et al.*, 2017). PYE has negative effects on the intracellular and intercellular redistribution of iron. It can down-regulate *FERRIC REDUCTASE OXIDASE3* (*FRO3*), *NICOTIANAMINE SYNTHASE4* (*NAS4*), and *ZINC-INDUCED FACILITATOR1* (*ZIF1*) (Long *et al.*, 2010), encoding components for iron allocation, and the subgroup Ib *BHLH* genes (Pu and Liang, 2023). The *pye-1* loss-of-function mutant is chlorotic, presumably because PYE controls the proper redistribution of iron in a cell-specific manner in the root stele and vasculature (Muhammad *et al.*, 2022). bHLH Ib transcription factors up-regulate root iron uptake, but they are only able to do so together with the subgroup IIIa bHLH transcription factor FER-LIKE IRON DEFICIENCY-INDUCED TRANSCRIPTION FACTOR (FIT). Together, they form an active nuclear transcription factor complex that up-regulates iron acquisition (Yuan *et al.*, 2008; Wang *et al.*, 2013; Trofimov *et al.*, 2019). FIT has been found to be responsive to environmental and hormonal cues as it integrates various signaling pathways to adjust iron acquisition accordingly, as discussed in Brumbarova *et al.* (2015) and Gratz *et al.* (2019). Genetic studies have been very informative in dissecting regulatory networks and dividing iron deficiency response genes into co-regulated gene expression networks that can be distinguished by their regulatory expression patterns (Fig. 2B) (Schwarz and Bauer, 2020; Schwarz *et al.*, 2020). The FIT-dependent ‘blue’ group comprises genes for iron acquisition and transport from soil into roots. ‘Blue’ components are induced in response to iron deficiency in seedling roots, requiring FIT. The FIT-independent ‘red’ group of genes and proteins is induced in response to iron deficiency in roots and shoots of seedlings and switched on in the absence of a functional FIT. These genes include targets of bHLH IVb and IVc factors (Schwarz and Bauer, 2020). ‘Rose-colored’ genes that activate iron deficiency are the above-mentioned *BHLHIVc* and *BHLHIVb* genes, and *BHLH11* that is up-regulated by sufficient iron. These genes are expressed in seedling roots and shoots and encode the upper regulatory level of the iron deficiency cascade (Fig. 2B). Some genes are down-regulated under iron deficiency. They include genes encoding components for iron storage and chelation or components that prevent circulation of excessive iron. Typical target genes in this ‘iron sufficiency’ group are the ferritin gene *FER1* and genes encoding nicotianamine synthase and YELLOWSTRIPE-LIKE transporters for translocation of nicotianamine–iron complexes (*NAS3*, *YSL1*, and *YSL3*). *FER1* is negatively controlled by the bHLH IVb transcription factor ILR3 (Tissot *et al.*, 2019).

The concept of a hierarchical organization of iron deficiency target genes and a discrimination into subnetworks and co-expression clusters (‘blue’, ‘red’, and ‘rose’ iron sufficiency clusters) has been very helpful for integrating new genetic components into the network and assessing positive and negative effects of the new components (Kim *et al.*, 2019; Lichtblau *et al.*, 2022; Muhammad *et al.*, 2022). This same concept can also become instrumental to decode light signaling cues that interfere with the process of iron deficiency response regulation.

## Influence of light and the circadian clock on iron uptake

Leaves play major roles in light perception and in the regulation of iron homeostasis, thereby offering potential cross-control. Iron is transported towards the shoot inside the xylem, and hence the transpiration stream is of prime relevance to iron allocation. Stomatal opening and transpiration depend on guard cell signaling, a process directly stimulated by blue and red light (Shimazaki *et al.*, 2007; Inoue and Kinoshita, 2017). Leaf xylem differentiation directly affects iron homeostasis as it is also relevant for water conductivity (Eichert *et al.*, 2010). Importantly, the leaf vasculature contains the tissues that respond first to an iron deficiency stimulus (Khan *et al.*, 2018). Hence, light-mediated control of iron homeostasis can be exerted in leaves.

**Fig. 3.**
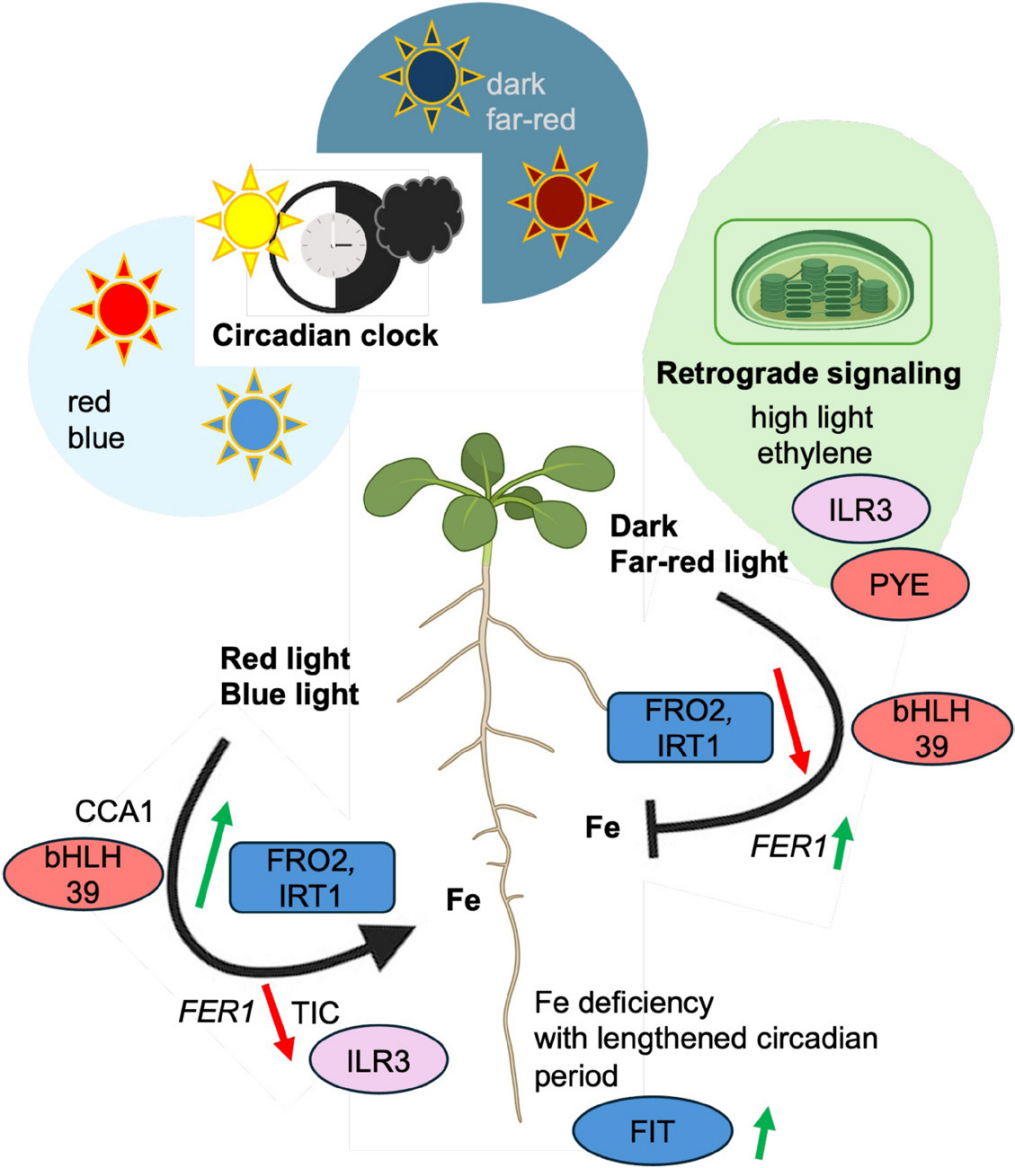
Light-dependent regulation of iron homeostasis. Different light conditions or darkness can influence iron acquisition and allocation positively or negatively, as indicated by black arrows. Red and blue light, in contrast to darkness and far-red light, can up- and down-regulate genes as represented by green and red arrows. The color code of iron deficiency components is as in Fig. 2. The circadian clock regulates iron homeostasis. Iron deficiency results in lengthening of the circadian period. The circadian clock components, CCA1 (CIRCADIAN CLOCK ASSOCIATED1) and TIC (TIME FOR COFFEE), may directly interfere with promoters of iron deficiency response genes or the iron sufficiency marker *FER1* (*FERRITIN1*). Retrograde signaling in chloroplasts, high light, and ethylene may intersect and control iron homeostasis. References are mentioned in the text. The figure has been created with Biorender.com.

Light stimulates iron deficiency response genes such as *BHLH39*, *FERRIC REDUCTASE-OXIDASE2* (*FRO2*), and *IRON REGULATED TRANSPORTER1* (*IRT1*) and root iron reductase activity, while at dawn expression of ferritin genes such as *FER1* increases (Vert *et al.*, 2003; Duc *et al.*, 2009; Trofimov *et al.*, 2024) (Fig. 3). *FIT* is an exception as it is regulated differently from ‘blue’ and ‘red’ genes. FIT is also induced by low iron in darkness (Trofimov *et al.*, 2024). In continuous darkness, on the other hand, etiolated leaves can still acquire iron (Sági-Kazár *et al.*, 2023). Studies on the nature of the mechanism controlling light-induced iron homeostasis have indicated that photoreceptor-mediated signaling, the circadian clock, and chloroplast functionality can be involved (Tissot *et al.*, 2014).

Different photoreceptors enable plants to perceive differing light spectra during the day and season (Paik and Huq, 2019). The circadian clock plays a crucial role in plants in coordinating various physiological and developmental processes in response to daily and seasonal changes in the environment (Xu *et al.*, 2019). Circadian rhythms control thousands of genes in *A. thaliana* (Tissot *et al.*, 2014). The circadian clock also synchronizes iron acquisition and iron allocation with the external day–night cycle, and hence is fundamental for optimizing iron resource allocation during growth (Fig. 3). The circadian clock of *A. thaliana* has two types of central components that form a double-negative feedback loop (Davis *et al.*, 2022). These are on one hand a set of morning components, MYB transcription factors CIRCADIAN CLOCK ASSOCIATED1 (CCA1) and LATE ELONGATED HYPOCOTYL (LHY1), and on the other hand a set of evening components, TIMING OF CAB EXPRESSION1 (TOC1) and PSEUDO-RESPONSE REGULATOR (PRR) proteins. CCA1/LHY1 and TOC1/PRR bind to and negatively regulate each other’s promoters. The stability of these proteins is controlled through protein–protein interaction, protein phosphorylation, and degradation via the proteasome. The continued feedback regulation ensures that the protein activities are oscillating in a circadian rhythm (Davis *et al.*, 2022). Promoter activities of *IRT1*, *BHLH39*, and *FER1* depend on clock components. *FER1* and other iron excess genes are under negative control exerted by the nuclear regulator and clock component TIME FOR COFFEE (TIC), as are downstream responses such as iron accumulation and ferritin protein formation. The promoter activities of *IRT1*, *BHLH39*, and *FER1* cycled together with those of *TOC1* and *CCA1* (Hong *et al.*, 2013). Interestingly, the circadian period was lengthened under iron deficiency compared with sufficiency (Chen *et al.*, 2013; Hong *et al.*, 2013). Under iron-deficient conditions, clock components also showed a lengthened circadian period. In addition to CCA1 and LHY, this observation was made for PRR5, PRR7, PRR9, GIGANTEA (GI), TOC1, and the nuclear protein and clock component EARLY FLOWERING4 (ELF4) (Salomé *et al.*, 2013). Additionally, the lengthened circadian period showed dependency on the amount of supplied iron by being longest when iron was least available and shortest under moderate iron supply (Salomé *et al.*, 2013). Other tested metal ions such as zinc, copper, and manganese did not influence the circadian period length in this way (Chen *et al.*, 2013; Salomé *et al.*, 2013). Hence, it is beneficial for plants to prolong iron acquisition during the day when iron supply is limited. It has also been described that expression of iron deficiency response genes is directly controlled by CCA1 binding to the respective iron deficiency response promoters (Xu *et al.*, 2019). The fact that iron acquisition and allocation depend on the circadian clock raises questions as to whether crops can be further improved in their abilities to use iron efficiently by better synchronizing the utilization of iron with day length.

Chloroplasts represent a major iron sink with 80% of total cellular iron content (Fig. 3). Lack of iron affects PSI, PSII, the cytochrome *b*_6_*f* complex, and ferredoxin, and the major phenotype is leaf chlorosis under iron-deficient conditions (Nouet *et al.*, 2011; Hantzis *et al.*, 2018). Iron excess can lead to oxidative damage as the photosynthetic electron transport chain is the source of ROS that can react with iron (Conte and Walker, 2011). Chloroplasts must have a way to signal iron status. In leaves, *FER1* is negatively controlled by bHLH subgroup IVc transcription factors, such as ILR3 and PYE, while the same transcription factors control iron distribution in leaves and up-regulate iron deficiency responses (Tissot *et al.*, 2019). They are also relevant for photoprotection upon high light intensity by preventing oxidative stress in leaves (Akmakjian *et al.*, 2021). Hence, there is a connection between high light, ROS, and iron responses in leaves.

Retrograde signaling may control iron homeostasis in leaves. This term refers to communication pathways that inform the nucleus about the metabolic state of organelles generating energy (chloroplasts and mitochondria) to influence nuclear gene expression. This can be achieved by metabolites produced in the organelles, that are translocated towards the cytosol and nucleus and ultimately interfere with the control of gene expression. One potential retrograde signal is the phosphonucleotide 3ʹ-phosphoadenosine 5ʹ-phosphate (PAP) which accumulates under stress such as drought and high light. PAP levels are negatively controlled by the enzyme SAL1, located in chloroplasts and mitochondria (Estavillo *et al.*, 2011). PAP may move from the chloroplast to the cytoplasm or nucleus to inhibit 5ʹ–3ʹ exoribonucleases (XRNs), which then can cause stress-related mRNAs to increase (Estavillo *et al.*, 2011). *Arabidopsis thaliana sal1* and *xrn4* mutant plants had enhanced levels of iron along with increased *FER1*, *FER3*, and *FER4* transcript amounts and signs of different post-transcriptional regulation of iron deficiency components (Estavillo *et al.*, 2011). The iron uptake machinery was also increased in the mutants, suggesting that components of the PAP/SAL1 retrograde signaling pathway control iron homeostasis (Balparda *et al.*, 2020, 2021). The cytosolic XRN4 (also known as ETHYLENE INSENSITIVE5, EIN5) is required for ethylene signaling (Potuschak *et al.*, 2006). Interestingly, the photoprotection gene *EARLY LIGHT-INDUCED PROTEIN* (*ELIP*) is up-regulated in *sal1* and *xrn4* mutants (Estavillo *et al.*, 2011). *ELIP* is also up-regulated in iron-deficient seedlings with a functional EIN3/EIN3-LIKE (EIL)1 as compared with *ein3 eil1* mutants (Bauer and Blondet, 2011; Lingam *et al.*, 2011). *ein3 eil1* mutants have iron deficiency response phenotypes, that had suggested that perhaps one role for EIN3/EIL1 might be to enhance iron acquisition to prevent oxidative stress under iron deficiency (Lingam *et al.*, 2011). It had been speculated that ethylene may promote iron acquisition as one way to deal with oxidative stress (Lingam *et al.*, 2011). Ethylene may act to enhance iron acquisition through signaling in leaves, potentially involving components of the PAP/SAL1 pathway. Finally, it should be noted that XRN4 is linked with circadian rhythm, and removing a functional XRN4 causes a lengthening of the circadian period, similar to under iron deficiency (Litthauer *et al.*, 2018). Hence, it is also a possibility that the effects of retrograde signaling and the circadian clock merge to control iron deficiency responses.

Furthermore, iron-related signals can be linked with heme- or nitric oxide-derived metabolites (Shekhawat and Verma, 2010; Santos *et al.*, 2019; Gracheva *et al.*, 2022; Romera *et al.*, 2023). Etiolated leaves are also competent to acquire iron and signal iron-related cues (Sági-Kazár *et al.*, 2022, 2023). Etiolation of green plants may also liberate iron stores.

Taken together, multiple leaf, chloroplast, and light signals are linked with iron signaling. More detailed studies are needed to better resolve how the various potential signaling routes converge and which concrete iron signaling molecules directly or indirectly control iron acquisition. One possibility to start with could be to better assess the circadian responses of iron allocation mutants and dissect the capabilities of individual leaves to act as sinks and sources for iron allocation and root iron uptake.

## Long-distance iron signaling

**Fig. 4.**
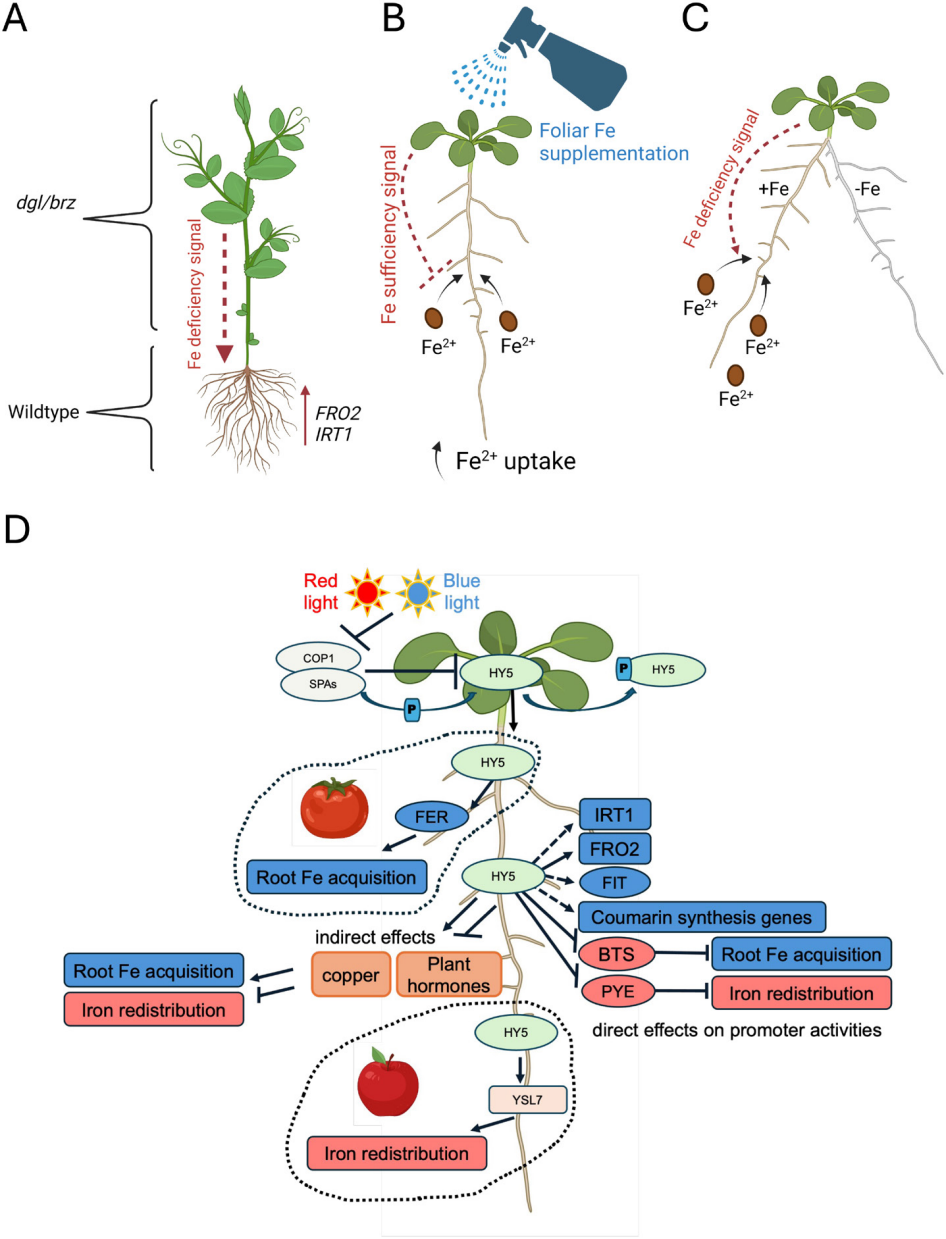
Long-distance signaling of the iron status, indicating the presence of diverse systemic iron signals. (A–C) Examples of three methods employed to reveal the importance of systemic shoot-to-root iron signals. (A) Reciprocal grafting using leaf iron-overaccumulating mutants such as *brz* (*bronze*) and *dgl* (*degenerate leaflet*) of pea (*Pisum sativum*) showed the existence of a long-distance shoot-to-root iron deficiency signal constitutively promoting root iron uptake and allocation towards leaves. (B) Foliar iron application to iron-deficient plants leads to a down-regulation of root iron uptake responses due to iron sufficiency signals. However, foliar application does not rescue the pea *dgl* and *Arabidopsis thaliana opt3* mutant defective in OLIGOPEPTIDE TRANSPORTER3, a component involved in the transmission of the long-distance iron sufficiency signal in the phloem. (C) Split root assays have demonstrated that iron uptake can be increased in response to local and systemic iron deficiency signals in root halves exposed to iron but not in root halves maintained in iron deficiency conditions. (D) HY5 (ELONGATED HYPOCOTYL5) is a regulator of iron homeostasis in roots. Light leads to activation of various photoreceptors, for example in response to red and blue light. Active photoreceptors inhibit the COP1 (CONSTITUTIVE PHOTOMORPHOGENIC1)–SPA (SUPPRESSOR OF PHYTOCHROME A) complex, thereby preventing degradation of HY5. A small pool of HY5 remains phosphorylated under the activity of SPA and is inactive even in the light. PPK1 is a photoregulatory protein kinase acting upon HY5. Active non-phosphorylated HY5 accumulates in light conditions and acts as a mobile signal in roots to induce various responses related to iron, as shown by studies in *A. thaliana*, tomato (*Solanum lycopersicum*), and apple (*Malus baccata*). In *A. thaliana*, HY5 can directly control expression of some genes for iron acquisition and allocation. Alternatively, HY5 may also indirectly control iron homeostasis as a consequence of its functioning in hormone and copper homeostasis. In tomato, HY5 can interfere with the action of FER, an ortholog of FIT. In apple, HY5 acts on a YELLOWSTRIPE-LIKE transporter gene (*YSL7*). References are mentioned in the text. The figure has been created with Biorender.com.

Root physiology can be dependent on systemic shoot-derived and light signals, and this provides a possibility for how light can affect iron uptake. This became evident from studying plant mutants affected in long-distance shoot-to-root regulation of iron acquisition (Fig. 4). Some of these mutants accumulate iron in the root stele or vasculature, but the high amounts of iron seem not to be readily bioavailable inside the plant to be allocated to various tissues (Rogers and Guerinot, 2002; Schuler *et al.*, 2012; Nguyen *et al.*, 2022; Chia *et al.*, 2023). As a consequence, such mutants may experience iron deficiency despite iron overload, and display deregulated iron signaling. Root iron uptake can be promoted through shoot-to-root long-distance iron deficiency signaling, as revealed through grafting experiments, for example with the pea *degenerate leaves* (*dgl*) and *bronze* (*brz*) mutants, probably carrying mutations in *OLIGOPEPTIDE TRANSPORTER3* (*OPT3*) and *BTS* genes (Welch and Larue, 1990; Grusak and Pezeshgi, 1996; Harrington *et al.*, 2024) (Fig. 4A). Foliar iron application experiments provided evidence for an iron sufficiency signal acting shoot to root and being transmissible via OPT3 (Garcia *et al.*, 2013) (Fig. 4B). Split-root experiments with one-half of the root system being exposed to iron-rich media, and the other half to iron-depleted conditions demonstrated that the need for iron mobilization is sensed both systematically and locally. Iron was preferentially mobilized in the root halves exposed to iron in the split-root situation while iron acquisition responses were abolished on the iron-deficient root halves (Vert *et al.*, 2003; De Nisi *et al.*, 2012). These experiments indicated that both root sides had been responsive to systemic iron deficiency signals while the presence of iron in the rhizosphere had a positive effect on mobilization (Fig. 4C). Finally, leaf excision experiments supported that leaves, but not the shoot apex, provided systemic information to instruct roots in mobilizing iron (Enomoto *et al.*, 2007). In sum, root iron uptake can be suppressed by shoot-derived long-distance iron sufficiency signals, while it can also be induced by long-distance iron deficiency signals. The exact nature of the systemic iron signals still needs to be revealed. *OPT3* is among the earliest iron-responsive genes, responding with an altered level of expression in the leaf phloem within 2–4 h of iron deficiency or iron resupply in comparison with roots which only respond from 24 h onwards (Zhai *et al.*, 2014; Khan *et al.*, 2018). IMA small proteins may be mobile signals to promote iron acquisition in roots (Grillet *et al.*, 2018; Tabata *et al.*, 2022). Recently, the ELONGATED HYPOCOTYL5 (HY5) transcription factor came to attention. HY5 is a shoot-to-root mobile protein that may control iron acquisition as a long-distance signal in the light.

### HY5 as a long-distance regulator for iron uptake

HY5 is activated by light signaling in the shoot and up-regulates light-mediated developmental processes, also in roots, and is linked with iron and micronutrient supply. In the absence of HY5, seedlings display elongated hypocotyls, a typical etiolation response (Oyama *et al.*, 1997). HY5 also regulates processes related to nutrient acquisition (Mankotia *et al.*, 2024). An example for that is the blue-light-controlled primary root growth inhibition in response to phosphate starvation, which includes iron accumulation at the tip, a response partly dependent on cryptochrome photoreceptors and HY5 (Gao *et al.*, 2021). This same example also indicates a positive effect of HY5 and blue light on nutrient and iron allocation in the root. Furthermore, different scenarios can explain how HY5 can affect iron nutrition. In the following, we introduce HY5 and summarize evidence that both direct and indirect effects speak in favor of HY5-promoted iron acquisition.

HY5 is a basic-leucine zipper (bZIP) transcription factor. HY5 protein stability is light regulated and its abundance is closely linked with the extent of photomorphogenesis (Osterlund *et al.*, 2000). *HY5* mRNA levels rise in response to light exposure, and the protein levels are further controlled post-translationally (Fig. 4D). There are typically two isoforms of HY5, phosphorylated and unphosphorylated. Studies have shown that the non-phosphorylated mutant form of HY5 has a higher binding to its target promoters, and higher transcriptional activity as compared with the phospho-mimicking mutant of HY5. The non-phosphorylation mutant also showed enhanced photomorphogenesis as it was found to have shorter hypocotyl length as compared with the wild type (Hardtke *et al.*, 2000; Wang *et al.*, 2021). Thus, the non-phosphorylated version of HY5 is the physiologically most active form for regulating photomorphogenesis, and perhaps it may also be the one that controls the regulation of nutrient homeostasis (Fig. 4D). HY5 can be phosphorylated by photoregulatory protein kinase PPK1 that has been recently described (Zhang *et al.*, 2024). By controlling the phosphorylation status of HY5, the activity of downstream targets of HY5 are fine-tuned, and HY5 levels are controlled through ubiquitination mediated by SUPPRESSOR OF PHYTOCHROME A (SPA) proteins (Wang *et al.*, 2021) and CONSTITUTIVE PHOTOMORPHOGENIC1 (COP1) protein, which are negative regulators of photomorphogenesis under dark conditions (Osterlund *et al.*, 2000; Wang *et al.*, 2021). The nuclear levels of the COP1–SPA complex are negatively regulated by light, which is mediated by the activity of multiple photoreceptors in response to various light wavelengths, thus allowing HY5 accumulation under light (Podolec and Ulm, 2018) (Fig. 4D). Since light is needed for iron acquisition to occur in roots, it can be assumed that the regulatory mechanisms that control HY5 also control iron uptake. To date, experimental evidence is lacking as to whether the photoregulatory protein kinases or the COP–SPA complex affects iron nutrition in response to iron deficiency. A study based on gene co-expression analysis suggested that PHYTOCHROME-INTERACTING TRANSCRIPTION FACTOR4 (PIF4) and HY5 might be involved in global transcriptome reprogramming to maintain iron homeostasis (Brumbarova and Ivanov, 2019).

One possibility is that HY5 acts directly upon the promoters of iron deficiency response genes (Fig. 4D). bZIP transcription factors such as HY5 are characterized by a basic region which enables DNA binding and an adjacent leucine zipper allowing bZIP dimerization. Interestingly, bZIP transcription factor-binding motifs are enriched in the FIT-dependent and -independent (‘blue’ and ‘red’) target gene promoters like those of bHLH transcription factors (Schwarz *et al.*, 2020). The presence of putative *cis*-regulatory elements suggests that bZIP proteins such as HY5 could indeed be involved in controlling expression of iron deficiency target genes. A recent study has revealed the role of HY5 in regulation of iron deficiency response in *A. thaliana*. A *hy5* mutant showed impaired growth and increased chlorosis under iron deficiency conditions as compared with the wild type (Mankotia *et al.*, 2023). The expression of ‘blue’ genes involved in iron uptake such as *FIT*, *IRT1*, *FRO2*, and coumarin biosynthesis genes were found to be positively regulated by HY5. *FRO2* was shown to be directly regulated by HY5. On the other hand, *BTS* and *PYE* were found to be directly negatively regulated by HY5 (Mankotia *et al.*, 2023). HY5 can also regulate iron uptake in tomato (*Solanum lycopersicum* L.). Red light activates SlHY5 through phytochrome B (PHYB); SlHY5 then can move from shoot to root and activate iron uptake by activating *SlFER* expression (Guo *et al.*, 2021). *SlFER* is the ortholog of *AtFIT* (Ling *et al.*, 2002). In apple (*Malus baccata*), MbHY5 may alleviate iron deficiency-induced chlorosis and promote iron transport by positively regulating the expression of *MbYSL7*, encoding a prospective nicotianamine–metal ion chelate transport protein (Sun *et al*., 2022). Hence, it is possible that HY5 acts as a long-distance signal. It may be translocated inside the phloem from shoot to root and directly control the promoter activities of iron deficiency response genes that contain bZIP-binding sites.

Another possibility is that HY5 regulates nutrient or hormone signaling pathways and thereby indirectly affects iron nutrition (Fig. 4D). HY5 lacks its own activation or repressor domain and its ability to activate or repress transcription depends on the transcription factor with which it interacts (Burko *et al.*, 2020). Several studies have highlighted the crucial involvement of HY5 in the regulation of hormone signaling pathways. Several hormone signaling pathways such as those involving auxin, gibberellic acid, brassinosteroids, and ethylene can be suppressed by HY5 (Sibout *et al.*, 2006; Lee *et al.*, 2007; Weller *et al.*, 2009; Li *et al.*, 2011; Shi *et al.*, 2011; Li and He, 2016). HY5 promotes abscisic acid (ABA) signaling and regulates the crosstalk between light and ABA signaling by physically interacting with ABA-INSENSITIVE5 (ABI5) (Chen *et al.*, 2008; Bhagat *et al.*, 2021). It is of interest to note that the various processes controlled by HY5 also indirectly intersect with the regulation of iron acquisition. For example, light has a positive effect on iron acquisition. Hormones such as auxin, ethylene, and gibberellic acid can regulate iron uptake via affecting expression of *FIT*, *FRO2*, and *IRT1* (García *et al.*, 2010; Lingam *et al.*, 2011; Giehl *et al.*, 2012; Brumbarova *et al.*, 2015; Wild *et al.*, 2016; Kanwar *et al.*, 2021). ABA alleviates iron deficiency stress by promoting the transport and reutilization of iron (Lei *et al.*, 2014; Zhang *et al.*, 2020). Brassinosteroids can negatively regulate iron deficiency responses (Wang *et al.*, 2012, 2015; Kour *et al.*, 2021). Therefore, HY5 could be indirectly involved in controlling iron uptake via hormone pathways. Additionally, several studies have reported that iron accumulation is enhanced under copper deficiency, and vice versa (Bernal *et al.*, 2012; Waters and Armbrust, 2013; Kastoori Ramamurthy *et al.*, 2018; Kroh and Pilon, 2020; Rai *et al.*, 2021). HY5 positively regulates copper homeostasis by interacting with SQUAMOSA PROMOTER BINDING PROTEIN-LIKE7 (SPL7) (Zhang *et al.*, 2014; Araki *et al.*, 2018). SPL7 can negatively regulate the expression of some of the iron deficiency-responsive genes (subgroup Ib *BHLH* and *IMA*) under iron deficiency (Kastoori Ramamurthy *et al.*, 2018). Hence, HY5 might indirectly regulate some of the iron homeostasis genes.

Overall, HY5 can act in multiple circumstances to control iron deficiency responses by directly and/or indirectly regulating key genes involved in iron deficiency signaling pathways. Further studies are required to fully understand the role of HY5 in regulation of iron homeostasis. The role of HY5 in regulation of iron transport or storage is not clear yet. The effects of photoreceptors and COP1–SPA1 complexes on HY5 and downstream iron acquisition responses remain to be investigated. HY5 protein needs to be visualized in plant roots, and it is of interest to understand tissue-specific localization and regulatory aspects. Indirect and direct effects can be disentangled by dissecting the hormone- and copper-mediated responses via HY5.

## Biomolecular condensates containing transcription factors of light and iron signaling

Cryptochromes, phytochromes, and iron signaling components can accumulate in subnuclear foci termed biomolecular condensates, and these may represent regulatory hubs for cross-connecting the regulation of iron uptake in response to light. In plants, condensation of nuclear proteins can be triggered, among other factors, by light and temperature. Photobodies are the subnuclear structures for which this phenomenon was first observed and is best studied in the plant field (Pardi and Nusinow, 2021). Condensates form as a result of protein interaction. The ability of proteins to engage in condensates such as photobodies depends on the presence of certain protein domains and in particular on the presence of intrinsically disordered regions. These allow proteins to be multivalent and engage in protein aggregation so that large protein complexes consisting of primary and secondary interacting proteins can form and separate into a dense phase from the remaining less concentrated nucleoplasm during phase separation (Kim *et al.*, 2023). The coalescing proteins form biomolecular condensates (Emenecker *et al.*, 2021; Pardi and Nusinow, 2021). A number of different proteins in addition to the blue and red light perception system are detected in photobodies, including HY5 and PIF bHLH transcription factors, indicating that photobodies are locations of regulatory protein interaction assemblies that can serve protein stability control, and transcriptional and post-transcriptional control (Pardi and Nusinow, 2021). Depending on the intensity and quality of light and temperature, photobodies can vary from being numerous and small to becoming large subnuclear entities (Pardi and Nusinow, 2021; Willige *et al.*, 2024).

**Fig. 5.**
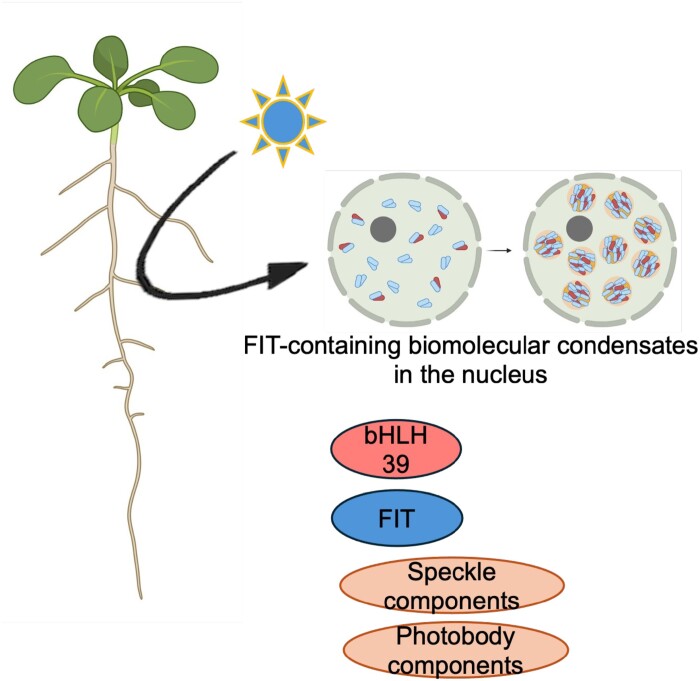
Blue light-dependent accumulation of iron deficiency response regulators in subnuclear biomolecular condensates. FIT and bHLH39 can form condensates, which are light inducible, reversible, highly dynamic, and probably formed due to liquid–liquid phase separation. The subnuclear bodies may contain speckle components, such as SR45 (ARGININE/SERINE-RICH45), and photobody components such as PIF (PHYTOCHROME-INTERACTING TRANSCRIPTION FACTOR) proteins. The figure has been created with Biorender.com.

It was recently found that an iron deficiency response regulator, namely FIT, can accumulate in biomolecular condensates in subnuclear membraneless compartments in the root epidermis of iron-deficient plants (Trofimov *et al.*, 2024). In several aspects, the FIT-containing nuclear bodies resembled photobodies. They were blue light inducible, reversible, highly dynamic, and the likely result of liquid–liquid phase separation (Fig. 5). FIT was able to form nuclear bodies containing active FIT–bHLH039 transcription factor complexes as well as homomeric FIT complexes (Trofimov *et al.*, 2024). FIT and bHLH subgroup Ib transcription factors have intrinsically disordered regions and undergo protein interactions in multiple ways via the N- or C-terminus or via their HLH domains (Yuan *et al.*, 2008; Wild *et al.*, 2016; Gratz *et al.*, 2019; Schwarz and Bauer, 2020; Lichtblau *et al.*, 2022). FIT also formed nuclear bodies with speckle components and splicing factors such as ARGININE/SERINE-RICH45 (SR45) (Trofimov *et al.*, 2024). This was interesting because mutation of SR45 splicing factor resulted in disturbed iron homeostasis. *sr45* mutants accumulated iron in the root stele, indicating that mutants were not able to use iron properly. This misutilization of iron might have been caused by aberrant iron deficiency response gene expression including differential alternative splicing of some iron response genes (Fanara *et al.*, 2022). Alternative splicing occurs in response to low iron (Li *et al.*, 2013; Fanara *et al.*, 2022; Trofimov *et al.*, 2024), and is also regulated by light, for example through phytochromes (Shikata *et al.*, 2014). FIT also co-localized with PIF3 and PIF4 bHLH factors in photobodies in particular dynamic patterns, indicating that FIT may be interwoven into photobodies without a blue light cue (Trofimov *et al.*, 2024). This behavior of FIT may speak in favor of a close association with PIF proteins in the nucleus (Fig. 5).

Thus, the presence of iron nutrition regulators in blue light-induced nuclear bodies indicates a cross-connection and complex signaling connecting light and iron deficiency signals (Fig. 5). Indeed, light-controlled iron nutrition-related nuclear bodies can be regulatory hubs in which multiple signaling pathways merge. There is clearly a need for future exploration to address the functional roles of protein complexes inside nuclear bodies with FIT protein for light and iron response regulation. The environmental factors for FIT condensation can be further elucidated to find out whether, for example, temperature and other abiotic signals can play a role.

## Conclusions

Light, in addition to nutrient availability, is a major environmental cue whose changes are directly registered by plants to redirect growth accordingly. The above analysis shows that light and iron nutrition signaling are interlinked in multiple ways. The concept of subdividing genes into ‘red’, ‘blue’, ‘rose’, and ‘iron sufficiency’ genes and understanding their regulatory principles is helpful in defining genetic and environment effects and consequences on iron nutrition. Clearly, future research can be intensified to better understand the cross-connection in iron and light signaling. Future endeavors can address the following as yet understudied open questions.

- How is transpiration controlled under low iron conditions? How does iron availability affect vascular differentiation to adjust iron nutrition to abiotic stress?
- How does etiolation affect iron acquisition? Etiolation can occur naturally during early plant development or appear as a consequence of a changing light environment during plant growth. What are the capacities of etiolated versus non-etiolated organs to act as iron sinks? Which effects does iron supply have on the capacities of etiolated versus non-etiolated leaves to acquire iron? Which signals are involved?
- How do changing light conditions, direction of light, shading, and varying light intensities during growth seasons affect the abilities of plants to utilize iron?
- How is internal iron supply controlled by iron signaling? Which sensing mechanisms are important? What are the roles of retrograde signaling via the chloroplasts and autophagy? How are these processes linked with high light regulation and the action of plant hormones, such as ethylene? Can other signaling molecules be relevant here, such as nitric oxide and heme?
- How are systemic leaf iron signals connected with light? What could be the nature of a systemic signal?
- How does HY5 signaling add specificity to the iron deficiency responses? How does HY5 signaling interact with the bHLH signaling controlling iron acquisition? Where is HY5 exerting effects in the root?
- What is the function of FIT nuclear bodies in iron signaling and which conditions favor their appearance? What is the role of alternative splicing of iron deficiency response genes? Is FIT forming protein interactions with light signaling components in roots? Are other iron deficiency response regulators also accumulating in subnuclear condensates following liquid–liquid phase separation?

With rising global temperatures and increasingly alkaline soils for agricultural purposes, we should experiment with and design novel crops. This will probably require improving nutrient use characteristics and modifying the photoperiod dependences so that novel crops can complete their life cycles in new environments. So far, the resulting effects of photoperiod changes on iron nutrition in different soils have been scarcely considered in crop improvements. Because of the need to develop sustainable crops with highly nutritious properties, more research should be dedicated to understanding and exploiting the interconnections between light signaling and iron nutrition.
